# Lead migration and postdural puncture headache following spinal cord stimulator placement in patient with cough: a case report

**DOI:** 10.1097/MS9.0000000000003104

**Published:** 2025-03-03

**Authors:** Edward Walton, Vladislav Pavlovich Zhitny, Brett Dixon, Ryan Jannoud, Ivan Rahman

**Affiliations:** a Department of Anesthesiology, Perioperative Care, and Pain Medicine, New York University, New York City, NY, USA; b Kirk Kerkorian School of Medicine, University of Nevada, Las Vegas, Las Vegas, NV, USA

**Keywords:** chronic pain management lead migration, postdural puncture headache, spinal cord stimulator

## Abstract

**Introduction and importance:**

Spinal cord stimulator (SCS) implantation has become a well-validated treatment for refractory chronic back pain. While generally safe, complications such as lead migration and postdural puncture headache (PDPH) can arise. Recognizing these hardware and biologic complications is crucial to improving outcomes and guiding management strategies.

**Case presentation:**

A middle-aged male with chronic nocturnal cough underwent successful percutaneous SCS trial placement under fluoroscopic guidance. Adequate pain coverage was confirmed, and he was discharged home. The following day, he returned with a positional headache, nausea, and imaging-confirmed lead migration. PDPH was suspected. At the time of presentation, a chest X-ray revealed possible lead migration, and the patient had also reported a chronic dry cough that had worsened the evening of the procedure. Management included removal of the leads and an epidural blood patch, leading to complete symptom resolution.

**Clinical discussion:**

Nocturnal coughing is hypothesized to have caused mechanical stress on the SCS leads, resulting in caudal migration. Lead migration is one of the most common SCS complications, with rates of 2.1–27%. Additionally, PDPH, though rare (~0.81% per lead placed), often requires an epidural blood patch for effective resolution. In this case, the displaced leads may have exacerbated an underlying dural tear, highlighting a novel interplay between hardware and biological complications.

**Conclusion:**

This is the first documented case at our academic center of concurrent lead migration and PDPH following SCS placement. Our findings suggest that chronic coughing may represent an unrecognized risk factor for lead migration and PDPH. Providers should address unresolved coughing prior to SCS implantation and consider improved anchoring techniques to minimize risks.

## Introduction

Over the past decade, spinal cord stimulator (SCS) implantation has become an increasingly popular and well-validated treatment modality for refractory chronic back pain. Although generally safe, SCS implantation carries potential hardware-related and biological risks that are worth recognizing. The overall complication rate is 30%^[1]^. Lead migration is the most frequently documented complication, with reported incidence rates ranging from 2.1% to 27% making it the most common reason for revision surgery following SCS implant^[2-6]^. A less-studied adverse outcome is postdural puncture and the subsequent development of postdural puncture headache (PDPH), with an incidence of about 1% per lead placed^[7]^.

**HIGHLIGHTS**

- First reported case of concurrent lead migration and PDPH after SCS placement.
- Nocturnal coughing linked to lead migration, a newly identified risk factor.
- PDPH risk is ~0.81% per lead; often needs an epidural blood patch to resolve.
- Vigilant follow-up and imaging key for headaches or reduced pain relief post-SCS.
- Better anchoring techniques and resolving cough may lower complication risk.

Lead migration is defined as an unintentional displacement of leads from their original surgical location, which can result in a loss of efficacy^[3]^. Recent studies suggest that the incidence of lead migration during SCS implantation varies widely with a range of 2.1–21%^[2-5]^. A retrospective analysis of 25 patients and 69 lead placements found an incidence of 78% significant lead migration during SCS trial with 94% of patients experiencing at least 1 lead migrating^[4]^. Similarly, an analysis of 91 SCS implantation cases found the proposed range of lead migration drastically underestimates lead displacement citing 88.5% of anchored leads migrated with or without clinical significance (i.e., loss of coverage)^[2]^. Although these studies do not agree with the current accepted definition of lead migration, the literature does imply that lead displacement after SCS implantation and during SCS trial may be more widespread than previously understood, though its clinical effects may be negligible.

Common risk factors for lead migration include higher BMI, specifically truncal obesity, vigorous postoperative activity, location of implantable pulse generator, and surgical technique^[2,3,7]^. We suspect that nocturnal coughing, as in our patient, may contribute to lead migration by producing increased mechanical stress on the leads. This hypothesis aligns with reports of intrinsic patient factors, such as elevated BMI, truncal obesity, and excessive postoperative activity in the setting of cough^[2,3,7]^.

Our case presents a unique intersection between hardware complications and a chronic dry cough, which has been linked to neural hyperexcitability. The patient’s cough persisted despite traditional management strategies, prompting consideration of pharmacological interventions. Pregabalin, a neural pathway inhibitor, has shown promise for treating resistant chronic cough by modulating sensory nerve hyperexcitability^[8]^. Such an approach might have alleviated the patient’s cough and mitigated mechanical stress on the SCS leads.

We present the first documented case at our academic center of concurrent lead migration and PDPH, as well as the first report linking these complications to a chronic cough. This work has been reported in line with the SCARE 2023 criteria^[9]^.

## Case presentation

**Figure 1.**
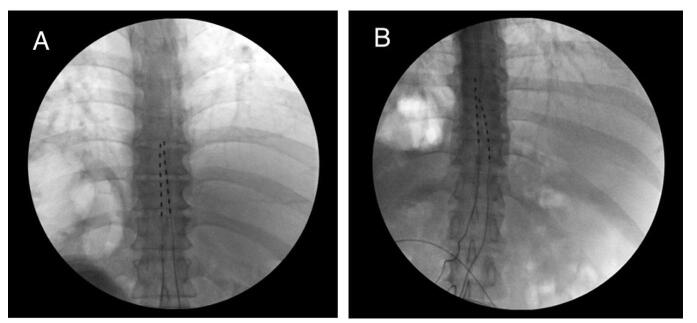
(A) AP radiographic view of initial lead placement with both leads at the superior endplate of T8. (B) AP radiographic view showing caudal migration of the right lead by 18 mm.

A middle-aged male with a history of depression, obesity, and failed back surgery syndrome of the lumbar spine status postphysical therapy, pharmacologic treatment, steroid injections, and two decompressive surgeries underwent percutaneous dual lead epidural spinal cord stimulator trial. During the procedure, a 14-gauge Tuohy needle trajectory was guided to the epidural space by a combination of loss of resistance technique and lateral/anterior posterior (AP) fluoroscopic imaging. No paresthesias were elicited and aspiration was negative for heme and cerebrospinal fluid (CSF). The leads were directed at the T8 vertebral body (Fig. 1A) and secured in place with benzoin and Steri-Strips. Electrical testing was performed to confirm satisfactory coverage of the patient’s pain.

At the time of SCS placement, the patient had been experiencing a chronic dry, nocturnal cough of unknown etiology for several months which was particularly severe in the evening immediately following the procedure. Overnight, the patient developed positional frontal–occipital headache, subjective fever, and nausea, prompting him to present to the emergency department the next morning. He denied change in pain coverage by the device. A chest X-ray performed in the ED revealed possible migration of a lead. He received intravenous fluids and caffeine without any alleviation in symptoms. Due to the likelihood of lead migration and PDPH, the decision was made to turn off the stimulator, remove the leads, and perform an L2/L3 epidural blood patch (EBP). AP radiograph at the time of EPB confirmed caudal migration of the right lead (Fig. 1B). The patient experienced subsequent complete alleviation of headache and was discharged home the same day. The clinical findings are presented in Table 1.

**Table 1:** Clinical findings throughout the duration of the procedure.

Clinical findings	Details
Patient background	Male with depression, obesity, and failed back
	Surgery syndrome
Procedure	Percutaneous dual lead spinal cord stimulator
	trial
Needle trajectory	Guided with fluoroscopy, placed at T8
Postprocedure symptoms	Cough, headache, fever, nausea
Chest X-ray	Possible lead migration
Management	IV fluids, caffeine, EBP
Outcome	Headache resolved, discharged same day.
Radiographic confirmation	Caudal lead migration confirmed

## Discussion

This case underscores the complexity of managing complications in SCS trials, particularly when hardware migration occurs concurrently with patient-specific factors such as chronic cough. Lead migration is a well-documented complication but is often underappreciated in its nuanced clinical presentations and potential triggers^[2]^. In our case, the nocturnal cough likely played an important role by exerting repeated mechanical stress on the leads, resulting in their caudal migration. This emphasizes the need to consider nontraditional mechanical risk factors, such as chronic respiratory symptoms, in patients undergoing SCS placement.

Additionally, this case illustrates the intersection of SCS hardware complications with neurological complications such as PDPH. The immediate resolution of PDPH symptoms following EBP suggests that the displaced leads may have caused a subtle dural tear, despite the absence of CSF during the initial procedure. This finding reinforces the importance of meticulous intraoperative technique, including careful attention to aspiration results and imaging, to minimize the risk of dural compromise. Additionally, it prompts further consideration of other preoperative measures, such as managing intrinsic risk factors like cough, obesity, or high BMI, to reduce the likelihood of complications.

Although permanent anchors were not utilized in our case, anchoring is a promising surgical practice that can potentially prevent lead movement after implantation. Mironer *et al* have developed an anchoring technique called “midline anchoring,” which significantly reduced loss of paresthesia coverage during a 4–5 day SCS trial when compared to a traditional epidural placement method^[10]^. More recently, a prospective study of patients undergoing SCS trial found a substantial difference in average lead migration between anchoring with tape alone vs tape and suture (8.72 mm vs 24.49 mm)^[11]^. In SCS implantations, some studies have found paddle lead implants (insulated laminectomy electrodes) have less lead migration, superior coverage, and improved short-term outcomes compared to percutaneous SCS lead placement^[12]^.

The incidence of PDPH in SCS placement is 0.81% per lead as described in a recent retrospective analysis^[7]^. The same study reported the lowest occurrence in the thoracolumbar region and the highest along the cervicothoracic spine^[7]^. A retrospective study with over 2000 patients found the incidence of unintentional dural puncture when obtaining access to the lumbar epidural space to be 0.5%^[1]^. Inadvertent dural puncture occurs in some cases due to difficulty distinguishing the epidural space on imaging, particularly the lateral view, which Shetty *et al* argues is because of overlying osseous structures and the shape of the epidural space itself^[1]^. One study further illustrated this concern and identified the contralateral oblique view as a potential tool for safely placing the needle near the lumbar epidural space prior to checking for LOR^[13]^.

Risk factors for PDPH development in SCS lead insertion include young age, low BMI, female sex, smaller epidural needle gauge size, lack of fluoroscopy or only a lateral fluoroscopic view^[7,14]^. PDPH is often self-limiting with 53% of patients finding spontaneous relief of pain within 4 days^[15]^. Conservative measures include bed rest, hydration, and pharmacotherapy, but these interventions often do not offer complete relief^[15]^. Current recommendations are for treatment of PDPH with EBP when supportive care fails or when symptoms become debilitating; this may help to curtail the risk of hospital readmission and permanent neurological sequelae^[16]^. Existing data indicate that patients who develop PDPH after undergoing SCS are generally unresponsive to conservative treatment and require EBP; this is anticipated given the 14-gauge needle size required for placement^[7]^. When performing an EBP in a patient with a permanent SCS, the decision to remove the leads is a clinical decision. One must weigh the complexity of lead placement as well as the feasibility of selecting another insertion site against the risk of infection when injecting autologous blood into an epidural space where there is existing implanted hardware^[7]^. Since our patient was only undergoing an SCS trial, we acted conservatively and removed the SCS entirely, ending the trial, prior to performing the EBP.

## Conclusion

Implanting providers must be familiar with complications that can arise in the subacute period following SCS placement. Though these complications are generally nonlife-threatening, they can potentially lead to loss of therapeutic efficacy, worse patient satisfaction, additional revision surgeries, and increased healthcare expenses. This case demonstrates that lead migration and dural puncture can occur simultaneously in patients with persistent coughing. Although not always feasible, we advocate waiting for resolution or improvement of cough prior to SCS placement to minimize possible risks. Further research is undoubtedly warranted in developing the safest and optimal SCS hardware and surgical techniques for implanting and securing leads. Meanwhile, clinicians should maintain a level of vigilance when examining SCS patients and have a low threshold for reimaging them, especially when presenting with headache, stimulation pattern changes and decreased pain control, or other neurological complaints.

## Data Availability

Data sharing is not applicable to this article.
